# Longitudinal Changes in Testicular Volume and the Additional Value of Body Weight for Estimating Histology-Linked Testicular Development in Male Cynomolgus Macaques (*Macaca fascicularis*)

**DOI:** 10.3390/ani16193113

**Published:** 2026-10-03

**Authors:** Ryosuke Kawashima, Hirofumi Matsumoto, Atsushi Asano

**Affiliations:** 1 Drug Safety Research Laboratories, Shin Nippon Biomedical Laboratories, Ltd., Kagoshima 891-1394, Japan; 2 Joint Faculty of Veterinary Medicine, Kagoshima University, Kagoshima 890-0065, Japan

**Keywords:** cynomolgus macaque, *Macaca fascicularis*, testicular volume, pubertal development, body weight, longitudinal analysis, geographic origin, reproductive toxicology, nonclinical safety study, animal selection

## Abstract

Individual variation in pubertal development limits the usefulness of chronological age alone for assessing testicular development in male cynomolgus macaques. This retrospective study characterized longitudinal changes in externally measured testicular volume and evaluated the additional information provided by body weight in Chinese- and Cambodian-origin animals. Routine records collected between 2006 and 2026 were analyzed. After data cleaning, 31,310 observations from 22,305 animals were available, including 4331 animals with repeated measurements. A prespecified mean bilateral testicular volume threshold of 8.68 cm^3^, previously associated with bilateral histological Grade ≥ 4, was applied. Testicular volume increased markedly between approximately 4 and 6 years of age, with substantial inter-individual variation. At 5 years, the estimated probability of attaining the threshold was 54.3% in Cambodian-origin and 51.6% in Chinese-origin animals, with no clear evidence of different age-dependent threshold trajectories between origins. In animals aged 2–8 years, body weight remained strongly associated with threshold attainment after adjustment for age and origin (OR 4.18 per kg, 95% CI 3.93–4.44; *p* < 0.001). These findings demonstrate substantial variation in testicular development among similarly aged animals and support direct testicular measurement as the primary practical screening measure, with age and body weight providing complementary information.

## Simple Summary

Male cynomolgus macaques are widely used in nonclinical safety studies in which reliable evaluation of the male reproductive system may be required. Chronological age is readily available when animals are selected for studies, but animals of similar age can differ markedly in testicular development. We retrospectively analyzed more than 31,000 routine testicular measurements from Chinese- and Cambodian-origin male cynomolgus macaques, including 4331 animals measured on more than one occasion. Testicular volume increased rapidly during approximately 4–6 years of age, but the timing of this increase varied among individuals. The longitudinal pattern was generally similar between geographic origins. Body weight was also strongly associated with testicular development after adjustment for age and provided additional information for identifying animals whose mean bilateral testicular volume was at or above 8.68 cm^3^, a previously identified screening value associated with a histological stage suitable for routine testicular toxicity evaluation. These findings indicate that chronological age alone does not fully describe individual testicular development. Direct testicular measurement remains the most biologically relevant practical indicator, while body weight provides useful additional information when selecting male cynomolgus macaques for studies requiring evaluation of the male reproductive system.

## 1. Introduction

Cynomolgus macaques (*Macaca fascicularis*) are widely used in nonclinical development, particularly for biopharmaceuticals for which the macaque may be the only pharmacologically relevant species. In this setting, the male reproductive tract is commonly evaluated within repeat-dose toxicity studies rather than in dedicated mating studies. Reliable interpretation of testicular histopathology therefore depends on the use of animals whose reproductive organs have reached a sufficiently advanced developmental stage [1,2]. Physiological changes in the seminiferous epithelium, germ-cell populations, epididymal sperm content, and accessory sex organs during puberty can otherwise resemble or obscure treatment-related findings [3,4,5,6].

Male reproductive development in cynomolgus macaques is a prolonged process rather than a discrete event. Classical developmental work showed gradual testicular growth during the prepubertal period followed by a pronounced increase around puberty [5]. Subsequent morphometric and histological studies demonstrated major changes in testicular weight, seminiferous tubular development, and spermatogenic cell composition during approximately 3–6 years of age [3,4,7]. Routine morphological staging further emphasizes that interpretation of the primate testis requires recognition of the normal cellular organization of spermatogenesis and its developmental state [8]. Consequently, chronological age is biologically informative but does not define an identical testicular status in every animal.

This inter-individual variability is important for practical study selection. Age and body weight have both been proposed as indirect indicators of male reproductive maturity [9]. Lower body weight has also been associated with features of late puberty even among animals otherwise considered sexually mature [6]. However, age and body weight primarily reflect chronological and somatic development, respectively, and neither directly measures the organ that will subsequently be evaluated histologically. Their usefulness is therefore likely to be probabilistic rather than deterministic.

External orchidometry provides a practical means of obtaining individual-level information about the testes before study assignment. Caliper-based measurements have been shown to distinguish immature from more developed male cynomolgus macaques [10], and later work confirmed the utility of orchidometric parameters for assessing sexual maturation [11]. Because the testes enlarge substantially as seminiferous tubules and germ-cell populations develop, testicular dimensions can serve as a biologically proximal marker of the underlying developmental process. Nevertheless, orchidometric measurements require interpretation in relation to a histologically meaningful endpoint rather than use of an arbitrary size criterion.

In our previously published histology-linked study, in-life testicular measurements were compared directly with bilateral testicular histology in 61 male cynomolgus macaques [12]. Mean bilateral testicular volume discriminated bilateral histological Grade ≥ 4 with an AUC of 0.976, and a ROC-derived cut-off of 8.68 cm^3^ provided 87.2% sensitivity and 95.5% specificity [12]. Grade ≥ 4 represented the earliest stage considered sufficiently developed for routine microscopic evaluation of spermatogenesis. The threshold was intended as a probability-based screening value rather than a definition of complete adult sexual maturity or fertility [12,13]. That study established the histological relevance and diagnostic performance of in-life testicular volume in a cross-sectional cohort, but it did not characterize how testicular volume changes longitudinally within individual animals, when animals enter the histology-linked screening range, whether these trajectories differ between major geographic origins, or whether body weight provides additional information in a large colony population.

Geographic origin may also contribute to variation because cynomolgus macaque populations used in biomedical research are genetically heterogeneous across regional populations [14,15,16], while husbandry and other environmental factors may contribute additional variation. However, previous developmental studies have largely relied on cross-sectional histological datasets or relatively limited orchidometric samples [3,4,5,7,9,10,11], making it difficult to distinguish persistent population differences from effects of age distribution, husbandry, or measurement schedule. Repeated routine measurements therefore provide an opportunity to characterize within-animal development while evaluating geographic origin as a potential modifier.

Accordingly, the present study had two primary objectives: (1) to characterize longitudinal age-related changes and inter-individual variability in externally measured testicular volume in male cynomolgus macaques; and (2) to determine whether body weight provides additional information beyond chronological age for identifying animals within the previously established histology-linked screening range. Geographic origin was additionally examined as a potential modifier of these relationships. We hypothesized that testicular volume would increase steeply during the pubertal period with substantial inter-individual variability and that body weight would provide additional, but not substitutive, information relative to direct testicular measurement.

## 2. Materials and Methods

### 2.1. Study Design and Data Source

This retrospective observational study used routine veterinary health-examination and colony-management records from male cynomolgus macaques maintained or evaluated by Shin Nippon Biomedical Laboratories, Ltd. Records collected from 11 January 2006 through 26 June 2026 were reviewed. The source dataset contained animal identification number, date of birth, geographic origin, date of testicular measurement, left and right testicular dimensions, date of body-weight measurement, and body weight. The present analysis focused on animals recorded as originating from China or Cambodia because these populations provided sufficiently large numbers of observations and repeated measurements for longitudinal analysis. The unequal numbers of Chinese- and Cambodian-origin animals reflected the composition and availability of animals in the historical colony database during the study period and did not result from prospective allocation or sampling by geographic origin.

Testicular and body-weight measurements were performed as part of routine veterinary health examinations and colony-management activities and were not conducted specifically for the present study. Routine testicular measurements were conducted according to an institutional standard operating procedure (SOP) reviewed and approved by the Institutional Animal Care and Use Committee (IACUC). No additional animal procedures, interventions, or manipulations were performed for this retrospective analysis. Accordingly, no study-specific IACUC protocol was generated for the present study.

### 2.2. Testicular Measurements and Volume Calculation

Testicular measurements were performed under ketamine sedation as part of routine physical examinations, using the standardized digital-caliper (ABS Solar Digimatic Caliper, Mitutoyo Corporation, Kawasaki, Japan) approach described previously [12]. The maximum longitudinal dimension and maximum transverse dimension of each testis were measured externally through the scrotal skin using digital calipers. All measurements were performed under the institutional SOP using standardized anatomical landmarks and the same digital-caliper method, and personnel performing the measurements received institutional training. Individual operator identifiers were not consistently retained throughout the approximately 20-year observation period; therefore, operator-specific variability could not be formally estimated or adjusted for.

Testicular volume was calculated separately for the left and right testes using the ellipsoid equation V = (π/6) × L × W^2^, where L represents testicular length and W represents testicular width. Measurements recorded in millimeters were converted to centimeters before calculation. Consistent with the animal-level approach used in the preceding histology-linked study [12], the arithmetic mean of the calculated left and right testicular volumes was used as the animal-level testicular volume for the primary analyses.

### 2.3. Histology-Linked Screening Threshold

A mean bilateral testicular volume of 8.68 cm^3^ was used as the primary screening threshold. This threshold was prespecified on the basis of our previously published, independent histology-linked cohort and was not derived or optimized using the present dataset [12]. In that study, 8.68 cm^3^ was the optimal ROC-derived cut-off for identifying animals with bilateral histological Grade ≥ 4, with an AUC of 0.976, sensitivity of 87.2%, and specificity of 95.5% [12]. Grade ≥ 4 represents the earliest histological stage at which the seminiferous epithelium is sufficiently developed for routine microscopic evaluation of spermatogenesis [3,4,12]. This threshold was therefore interpreted as a probability-based screening value associated with a histologically evaluable stage rather than as proof of complete adult maturity or fertility. The present study was not intended to re-estimate or revalidate the diagnostic performance of this threshold, but rather to apply the independently derived threshold to a large longitudinal historical dataset.

### 2.4. Body-Weight Measurements

Body weight was measured under routine husbandry conditions without prior fasting. Because body-weight measurements were not always performed on exactly the same date as testicular measurements, the absolute temporal difference between the two measurements was quantified for each record. Among 31,310 technically valid observations, 30,607 body-weight measurements (97.8%) were obtained on the same day as the corresponding testicular measurement; 30,934 (98.8%) were within ±7 days, 31,169 (99.5%) within ±30 days, 31,269 (99.9%) within ±90 days, and 31,300 (>99.9%) within ±180 days.

For the primary body-weight analysis, body-weight values obtained within ±30 days of the testicular measurement were accepted. Sensitivity analyses were performed using same-day measurements and windows of ±7, ±90, and ±180 days. When suitable body-weight measurements were available both before and after the testicular measurement, a supplementary analysis using linear interpolation was also performed. Results of the body-weight timing sensitivity analyses are summarized in Supplementary Table S3.

### 2.5. Data Cleaning and Outlier Handling

Among Chinese- and Cambodian-origin records, 34,302 source records were screened. Records lacking one or more dimensions required to calculate both left and right testicular volumes (*n* = 2776) and records containing zero or other non-positive testicular dimensions (*n* = 212) were excluded. Four additional records were excluded as obvious data-entry errors because a single recorded dimension was 85, 86, 313, or 385 mm, whereas the remaining three dimensions in the same record ranged from 8 to 43 mm. These exclusions were made before statistical modeling. The resulting technically valid dataset contained 31,310 observations from 22,305 individual animals. Exclusion of incomplete bilateral measurements was applied at the observation level rather than the animal level; animals remained eligible at other time points if valid bilateral measurements were available. A detailed accounting of records retained and excluded at each stage of data preparation, including technical validity checks, eligibility criteria, and statistical outlier exclusion, is provided in Supplementary Table S4.

For the primary body-weight analysis, observations were restricted to animals aged 2–8 years and to body-weight measurements obtained within ±30 days of testicular measurement. Statistical outliers were assessed separately for log-transformed body weight and log-transformed mean bilateral testicular volume using residuals from models adjusted for age and geographic origin. An observation was excluded if the absolute residual for either variable exceeded five median absolute deviations from the corresponding residual distribution. Seventeen observations met this prespecified statistical outlier criterion. The final primary body-weight dataset therefore contained 29,051 observations from 21,932 individual animals. Outlier exclusion was applied only to the primary body-weight analysis and did not affect the longitudinal testicular-volume analyses. As an additional sensitivity check, the primary longitudinal binary and continuous GEE models were refitted while retaining the four obvious data-entry errors.

### 2.6. Longitudinal Transition Analysis

Animals with two or more valid testicular measurements were identified for longitudinal analysis. A total of 4331 animals had repeated measurements, including 3072 Cambodian-origin and 1259 Chinese-origin animals. For descriptive transition analysis, animals whose first valid mean bilateral testicular volume was below 8.68 cm^3^ were followed across subsequent observations. The first measurement at or above 8.68 cm^3^ was defined as the first observed threshold attainment. Because the true biological transition occurred between measurement dates, the midpoint between the last below-threshold measurement and the first at-or-above-threshold measurement was additionally calculated as a descriptive estimate of transition age. This midpoint was used for descriptive purposes only and was not interpreted as the exact biological time of threshold attainment. The individual-level transition dataset is provided as the accompanying Supplementary Dataset (Animals_Article_Supplementary_Data.xlsx; sheet: Table S5_Transitions) and is described in Supplementary Table S5.

### 2.7. Statistical Analysis

Repeated measurements within the same animal were analyzed using generalized estimating equations (GEEs) with an exchangeable working correlation structure and robust standard errors [17]. GEEs were selected because the primary objective was to estimate population-averaged developmental associations and trajectories while accounting for within-animal correlation, rather than to obtain subject-specific developmental trajectories or individual predictions. Analyses of the principal developmental period were restricted to animals aged 2–8 years.

For the binary threshold analysis, whether mean bilateral testicular volume was ≥8.68 cm^3^ was modeled using a binomial distribution with a logit link. The longitudinal model included chronological age centered at 5 years (age_c = age − 5), the squared centered-age term (age_c^2^), geographic origin, and an age_c-by-origin interaction. The quadratic age term was included to accommodate the expected nonlinear pattern of testicular development during the pubertal period. Full coefficient estimates for the longitudinal binary GEE model are provided in Supplementary Table S1.

For continuous testicular volume, mean bilateral testicular volume was natural-log transformed and modeled using Gaussian GEEs with the same centered-age, squared centered-age, geographic-origin, and age_c-by-origin terms. Full coefficient estimates for the continuous-volume GEE model are provided in Supplementary Table S2.

As a sensitivity analysis of the functional form of age, the quadratic age specification was replaced with a restricted cubic spline within the same GEE framework [18,19]. Four knot locations corresponding to the 5th, 35th, 65th, and 95th percentiles of the observed age distribution were used (approximately 2.92, 4.46, 5.50, and 7.39 years, respectively). Geographic origin and a linear age-by-origin interaction were retained. This analysis was performed to assess whether the principal developmental pattern was robust to a more flexible representation of the nonlinear association with age. The corresponding sensitivity analyses are presented in Supplementary Figures S1 and S2.

To investigate the additional value of body weight, body weight in kilograms was added to the GEE models. The primary body-weight model included age_c, age_c^2^, body weight, geographic origin, and an age_c-by-origin interaction. A body-weight-by-origin interaction was examined in a secondary analysis. The apparent discrimination of models with and without body weight for identifying observations with mean bilateral testicular volume ≥ 8.68 cm^3^ was summarized using the area under the receiver operating characteristic curve (AUC). Because these AUC values were calculated in the analysis dataset, they represent apparent rather than externally validated predictive performance. Accordingly, differences in AUC between models were interpreted descriptively and were not treated as evidence of externally validated improvement in predictive performance.

Associations between age and body weight were assessed using Pearson and Spearman correlations, and multicollinearity was examined using variance inflation factors (VIFs). For descriptive comparison of threshold-transition proportions between origins, Pearson’s chi-square test with Yates’ continuity correction was used. Continuous transition measures were compared using the two-sided Mann–Whitney U test. Statistical significance was defined as *p* < 0.05. Analyses were performed usingR version 4.4.2 (R Foundation for Statistical Computing, Vienna, Austria)..

### 2.8. Bilateral Asymmetry Analysis

To address potential masking of unilateral differences by the bilateral mean, left–right asymmetry was characterized descriptively across all technically valid observations. The absolute volume difference was calculated as |V_left − V_right|; relative bilateral difference was calculated as |V_left − V_right| divided by the bilateral mean and expressed as a percentage; and the larger-to-smaller testicular-volume ratio was calculated. Because the 8.68 cm^3^ screening threshold was derived for mean bilateral volume, it was not reinterpreted as a unilateral diagnostic cutoff. To assess whether repeated measurements materially influenced the descriptive distribution, the analysis was repeated using only the first valid observation from each animal. Results are summarized in Supplementary Table S6.

## 3. Results

### 3.1. Dataset and Repeated Measurements

The flow of records from the source dataset to the final analytical datasets is summarized in Supplementary Table S4. After technical data cleaning, 31,310 valid observations from 22,305 Chinese- and Cambodian-origin male cynomolgus macaques were available. A total of 4331 animals had at least two valid testicular measurements. Of these, 3072 were of Cambodian origin and 1259 were of Chinese origin. Among repeatedly measured animals, 2739 had a mean bilateral testicular volume below 8.68 cm^3^ at their first valid observation. During subsequent follow-up, 1596 animals were observed to reach or exceed the threshold.

### 3.2. Longitudinal Attainment of the Histology-Linked Threshold

Among animals initially below the prespecified threshold, 1167 of 2018 Cambodian-origin animals (57.8%) and 429 of 721 Chinese-origin animals (59.5%) subsequently reached or exceeded the threshold. The observed transition proportions did not differ significantly between origins (*p* = 0.461).

The first observed age at which animals reached ≥8.68 cm^3^ was lower in Cambodian-origin animals than in Chinese-origin animals (median 5.29 years, interquartile range [IQR] 4.70–6.01 vs. 5.72 years, IQR 5.06–6.40; *p* < 0.001). However, measurement intervals differed substantially between populations. The median interval spanning the threshold transition was 0.66 years in Cambodian-origin animals and 1.18 years in Chinese-origin animals. When the midpoint between the last below-threshold and first at-or-above-threshold observations was used as a descriptive estimate, the median estimated transition age was 4.90 years (IQR 4.35–5.54) in Cambodian-origin animals and 5.07 years (IQR 4.56–5.62) in Chinese-origin animals. Although statistically significant, the absolute difference was approximately 0.17 years. These findings indicate that apparent differences in first observed threshold-attainment age were partly influenced by differences in measurement frequency (Table 1).

### 3.3. Age-Dependent Longitudinal Development and Geographic Origin

**Figure 1:**
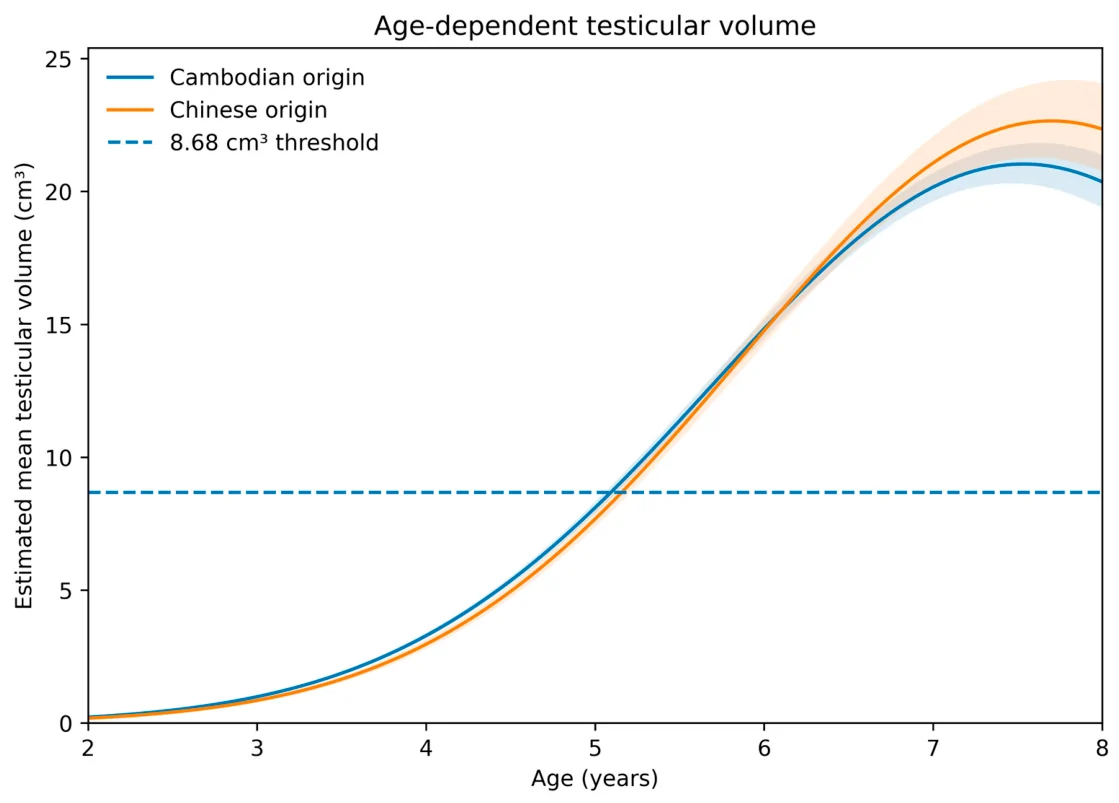
Age-dependent mean bilateral testicular volume estimated using Gaussian GEEs with log-transformed volume. Curves represent model estimates for Cambodian- and Chinese-origin male cynomolgus macaques aged 2–8 years; shaded regions indicate 95% confidence intervals. The dashed horizontal line indicates the previously identified 8.68 cm^3^ histology-linked screening threshold. The slight decline predicted near the upper age boundary reflects the quadratic functional form; it was not reproduced in the restricted cubic spline sensitivity analysis and should not be interpreted as biological regression of testicular volume.

**Figure 2:**
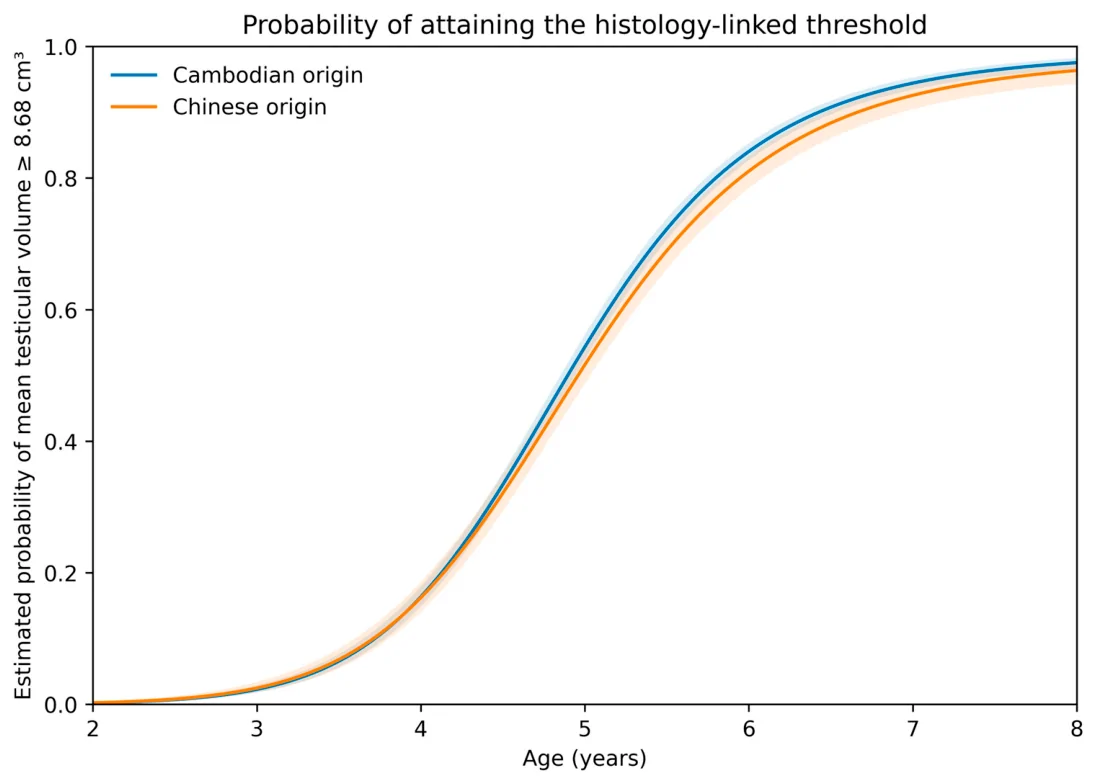
Age-dependent probability of mean bilateral testicular volume ≥ 8.68 cm^3^ estimated using binomial GEEs. Curves represent Cambodian- and Chinese-origin male cynomolgus macaques aged 2–8 years; shaded regions indicate 95% confidence intervals. The restricted cubic spline sensitivity analysis reproduced the overall age-dependent threshold trajectory. The slight upper-age decline predicted by the quadratic continuous-volume model (Figure 1) was not reproduced by the spline analysis and is considered a model artifact without biological meaning.

The repeated-measures GEE analysis of animals aged 2–8 years included 11,451 observations from 4195 animals. The probability of attaining the histology-linked screening threshold increased markedly with chronological age. Model-estimated probabilities for Cambodian-origin animals were 2.3% at 3 years, 16.3% at 4 years, 54.3% at 5 years, 84.0% at 6 years, and 94.4% at 7 years. Corresponding estimates for Chinese-origin animals were 2.5%, 16.2%, 51.6%, 81.0%, and 92.6%, respectively. In this model, neither the origin term (*p* = 0.097) nor the age-by-origin interaction (*p* = 0.160) was statistically significant, providing no clear statistical evidence that the age-dependent threshold trajectories differed between the two populations. Age-dependent changes in continuous testicular volume and threshold-attainment probability are shown in Figure 1 and Figure 2, respectively, and representative model estimates are summarized in Table 2.

Continuous testicular volume showed the same general developmental pattern (Figure 1; Table 2). Model-estimated geometric mean volumes for Cambodian-origin animals were 0.99, 3.29, 8.10, 14.83, and 20.16 cm^3^ at 3, 4, 5, 6, and 7 years, respectively. Corresponding values for Chinese-origin animals were 0.85, 2.97, 7.68, 14.76, and 21.07 cm^3^. Although origin-related terms were statistically significant in the continuous-volume model, absolute differences during the central pubertal period were small relative to the magnitude of the age-dependent increase. The slight downturn in predicted testicular volume near the upper age boundary reflects the quadratic functional form; it was not reproduced in the restricted cubic spline sensitivity analysis and should not be interpreted as biological regression of testicular volume.

### 3.4. Temporal Agreement Between Body-Weight and Testicular Measurements

Body-weight measurements were generally obtained close to the corresponding testicular measurements. Of 31,310 technically valid observations, 30,607 (97.8%) had body weight measured on the same day, 30,934 (98.8%) within ±7 days, 31,169 (99.5%) within ±30 days, 31,269 (99.9%) within ±90 days, and 31,300 (>99.9%) within ±180 days. Thus, temporal mismatch between testicular and body-weight measurements was minimal for the vast majority of observations.

### 3.5. Association Between Body Weight and the Histology-Linked Threshold

The primary body-weight analysis included 29,051 observations from 21,932 animals aged 2–8 years. After adjustment for chronological age, its quadratic term, geographic origin, and the age-by-origin interaction, body weight remained strongly associated with mean bilateral testicular volume ≥ 8.68 cm^3^. Each 1 kg increase in body weight was associated with an odds ratio of 4.18 for threshold attainment (95% CI 3.93–4.44; *p* < 0.001). The primary body-weight-adjusted model is summarized in Table 3. The body-weight-by-origin interaction was not statistically significant for the binary outcome (*p* = 0.060), providing no clear statistical evidence that the association between body weight and threshold attainment differed between origins.

**Figure 3:**
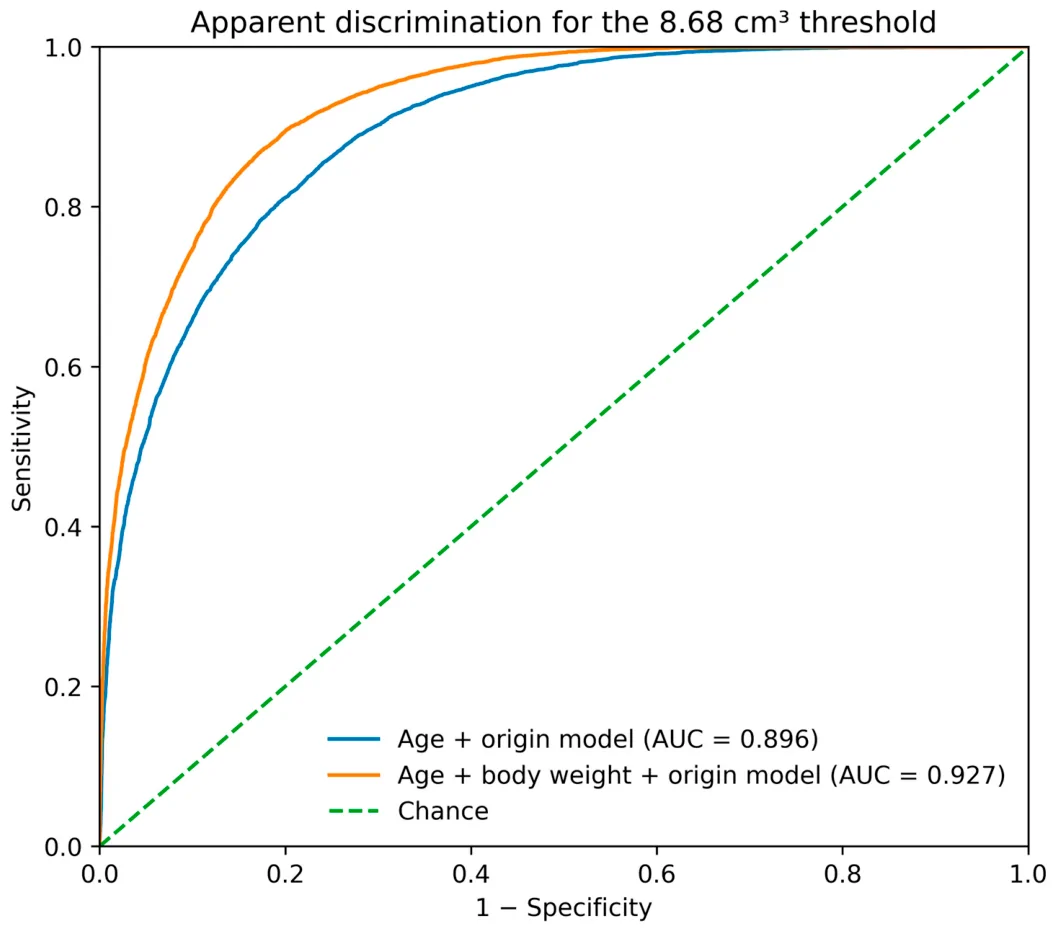
Receiver operating characteristic curves showing apparent discrimination of the age-plus-origin model and the model additionally including body weight for identifying observations with mean bilateral testicular volume ≥ 8.68 cm^3^. AUC values were calculated in the same analysis dataset used for model fitting and therefore represent apparent discrimination rather than externally validated predictive performance.

The apparent AUC was approximately 0.896 for the model based on age and origin and approximately 0.927 after inclusion of body weight, corresponding to an absolute increase of approximately 0.031 in apparent discrimination (Figure 3). Because the AUCs were estimated in the same dataset used for model fitting, this comparison was interpreted descriptively and does not constitute external validation of predictive improvement.

Age_c = chronological age − 5 years. The origin-related coefficients in the body-weight-adjusted model should be interpreted conditionally because age, body weight, and origin were correlated and an age-by-origin interaction was included. In contrast, the longitudinal model without body weight showed no clear origin difference in the age-dependent threshold trajectory.

### 3.6. Body Weight and Continuous Testicular Volume

Body weight was also independently associated with continuous testicular volume. In the log-volume GEE model, each additional kilogram of body weight was associated with a 1.62-fold higher mean bilateral testicular volume (95% CI 1.59–1.64; *p* < 0.001) after adjustment for age and origin. The root mean squared error for log-transformed testicular volume decreased from approximately 0.79 for the age-based model to approximately 0.70 after inclusion of body weight.

Chronological age and body weight were strongly correlated (Pearson r = 0.83; Spearman ρ = 0.85). Nevertheless, VIFs were approximately 4.33 for age and 3.31 for body weight, indicating moderate but not prohibitive multicollinearity. A body-weight-by-origin interaction was statistically significant for continuous testicular volume (multiplicative interaction estimate approximately 1.07; *p* < 0.001), suggesting a modest difference in the continuous body-weight–volume relationship between origins despite the broadly similar binary threshold association.

### 3.7. Sensitivity Analyses

The association between body weight and threshold attainment was highly stable when the allowable interval between body-weight and testicular measurements was varied. The estimated odds ratio per kilogram was 4.18 for same-day measurements and ranged from 4.16 to 4.19 across analyses using ±7-, ±30-, ±90-, and ±180-day windows. The estimated association between body weight and continuous testicular volume was similarly stable, with volume multipliers of approximately 1.62 per kilogram across all timing windows. Apparent AUC and RMSE estimates also changed minimally across the alternative measurement windows. These findings indicate that the small degree of temporal mismatch between body-weight and testicular measurements did not materially affect the principal conclusions. Detailed estimates across the alternative body-weight measurement windows are provided in Supplementary Table S3.

Sensitivity analyses using restricted cubic splines for age reproduced the principal developmental pattern observed in the quadratic GEE models. The estimated probability of attaining the 8.68 cm^3^ threshold increased markedly between approximately 4 and 6 years of age. At 5 years, the estimated probabilities were 55.0% for Cambodian-origin animals and 52.2% for Chinese-origin animals, increasing to 82.5% and 79.3%, respectively, at 6 years. The origin term and linear age-by-origin interaction were not statistically significant in the binary threshold model (*p* = 0.081 and *p* = 0.230, respectively). The continuous-volume spline model similarly reproduced the marked pubertal increase. However, unlike the quadratic model, the spline model did not predict a decline in testicular volume toward the upper end of the 2–8-year age range. These findings indicate that the principal conclusions regarding pubertal testicular development were robust to the functional form used to model age (Figures S1 and S2). Retaining the four obvious data-entry errors increased the longitudinal analysis dataset from 11,451 to 11,453 observations because only two of the four records contributed to the 2–8-year repeated-measures analysis. The largest absolute change in any fitted regression coefficient was <0.001 in both models, and the inferential conclusions were unchanged.

### 3.8. Bilateral Testicular-Volume Asymmetry

Across 31,310 technically valid observations, the median absolute difference between left and right testicular volumes was 0.386 cm^3^ (IQR, 0.075–1.283), and the median relative bilateral difference was 9.30% (IQR, 2.99–19.43%). The 95th percentile of the relative bilateral difference was 40.78%. The median larger-to-smaller testicular-volume ratio was 1.098 (IQR, 1.030–1.215); ratios ≥1.5, ≥2.0, and ≥3.0 occurred in 1626 (5.19%), 345 (1.10%), and 110 (0.35%) observations, respectively. Results were similar when the analysis was restricted to the first valid observation from each animal (*n* = 22,305), indicating that the distribution was not materially driven by repeated observations from a subset of animals (Supplementary Table S6).

## 4. Discussion

The present study provides a large-scale longitudinal characterization of external testicular development in male cynomolgus macaques and extends a previously established histology-linked screening approach to routine colony data. Three findings are particularly relevant. First, testicular volume increased steeply during approximately 4–6 years of age, with substantial inter-individual variation in the timing of this increase. Second, the age-dependent probability of attaining the histology-linked screening threshold showed no clear evidence of a major difference between Chinese- and Cambodian-origin animals when repeated measurements and age were taken into account. Third, body weight remained strongly associated with threshold attainment and continuous testicular volume after adjustment for age, indicating that somatic growth provides information beyond chronological age. Together, these findings support individual testicular assessment rather than reliance on a fixed age or geographic-origin criterion. Importantly, the present study was not designed to re-estimate or independently validate the diagnostic performance of the 8.68 cm^3^ threshold. Rather, it applied a prespecified threshold derived from animals with paired in-life testicular measurements and bilateral histopathology to characterize developmental patterns in a large longitudinal colony population. Because histopathological examination was not available for animals in the present historical dataset, threshold attainment should be interpreted as entry into a previously defined screening range associated with a histological stage suitable for testicular evaluation, rather than as direct histological confirmation of maturity in an individual animal. This distinction is consistent with broader NHP reproductive-safety practice, in which histopathology and organ-level endpoints remain central because dedicated functional fertility studies in NHPs are often impractical and individual reproductive endpoints may have limited sensitivity [20,21]. Careful morphologic interpretation is also essential because treatment-related testicular changes must be distinguished from normal spermatogenic architecture and background findings [22].

The longitudinal pattern observed here is consistent with the known biology of male macaque puberty. Kluin et al. described gradual testicular growth during early postnatal development followed by a marked pubertal expansion [5], while Haruyama et al. demonstrated age-related progression in testicular weight, seminiferous tubular morphology, and spermatogenesis in cynomolgus macaques [3,4]. The present analysis places these histological observations into an individual longitudinal framework. Model-estimated mean bilateral testicular volume remained small at approximately 3 years, increased substantially between 4 and 6 years, and was markedly larger by 6–7 years. The corresponding probability of reaching the histology-linked screening range increased from approximately 16% at 4 years to approximately 50% at 5 years and more than 80% at 6 years. Thus, pubertal testicular development is rapid at the population level but remains highly heterogeneous among individuals. The robustness of this developmental pattern was supported by restricted cubic spline sensitivity analyses, which reproduced the marked increase during approximately 4–6 years of age without requiring a quadratic age function. Notably, the slight decline in testicular volume toward the upper end of the age range predicted by the quadratic model was not reproduced by the spline model and is therefore interpreted as an artifact of the quadratic functional form without biological meaning. The biologically relevant interpretation concerns the marked pubertal increase and substantial inter-individual heterogeneity rather than postpubertal regression of testicular volume.

The substantial heterogeneity within age groups is particularly relevant for animal selection. At 5 years of age, approximately half of the animals had not yet reached the histology-linked screening threshold. Thus, even among animals of exactly the same chronological age, a substantial proportion may remain at a less developed testicular stage, whereas others have already entered a range previously associated with histological suitability for routine evaluation of spermatogenesis. A single chronological age cut-off would therefore classify animals with materially different testicular developmental states as equivalent. This finding is consistent with previous reports that age alone is an imperfect indicator of sexual maturation [9,11] and with the recognized difficulty of interpreting immature or peripubertal reproductive tissues in toxicity studies [1,2]. The present longitudinal dataset extends these observations by demonstrating substantial developmental variability within a very large population and across repeated measurements within individual animals.

Geographic origin produced a more nuanced result. In the descriptive transition analysis, the first observed threshold-attainment age was later in Chinese-origin animals than in Cambodian-origin animals. However, Chinese-origin animals also had longer intervals between measurements around the transition. When the midpoint of the interval between the last below-threshold and first above-threshold measurement was used as a descriptive estimate, the difference decreased to approximately 0.17 years. More importantly, the repeated-measures threshold model without body weight showed no clear origin effect or age-by-origin interaction on the developmental trajectory. These findings indicate that measurement schedule can create an apparent difference in observed transition age and that the biological magnitude of the origin difference is substantially smaller than would be inferred from the first positive measurement alone.

This interpretation is relevant because cynomolgus macaques are not a genetically homogeneous population. Genetic studies have demonstrated substantial population structure and differences in allelic diversity among source populations [14]. Geographic origin can therefore be biologically relevant in nonclinical research, and reproductive-tract findings have also been examined in relation to origin [6]. Nevertheless, ‘origin’ aggregates genetics, breeding history, nutrition, management practices, and other environmental factors. The present results do not exclude subtle population effects; rather, they show that origin alone is a poor surrogate for the developmental status of an individual male. For study selection, the within-origin and within-age variability is more consequential than the relatively small difference between the two population-level trajectories.

Body weight provided a second source of individual-level information. After adjustment for age and origin, each 1 kg increase in body weight was associated with approximately fourfold greater odds of attaining the histology-linked screening threshold. Body weight was also strongly associated with continuous testicular volume. These results are biologically plausible because somatic and reproductive development occur concurrently during adolescence and agree with earlier work identifying body weight as a predictor of testicular maturity [9] and with observations that lower-weight adult-classified males may retain late-pubertal features [6]. Importantly, the association persisted after inclusion of age in the model, indicating that body weight was not simply a numerical proxy for chronological age. Previous breeder-selection work likewise found that age, body weight, testicular volume, endocrine measures, and semen characteristics are most informative when interpreted together rather than as isolated criteria [23].

The increase in apparent discrimination after adding body weight—from an AUC of approximately 0.896 to 0.927—is a descriptive measure of incremental information within the present dataset and should not be interpreted as externally validated diagnostic improvement. The AUCs were calculated in the same dataset used for model estimation, and the outcome was defined using testicular volume itself rather than independent histology. Thus, the analysis demonstrates statistical information added by body weight for identifying the histology-linked volume range, not independent diagnostic performance for microscopic maturity. External validation against histology in a separate colony would be required before age-and-weight equations could be proposed as a screening algorithm.

Body weight also should not be viewed as a replacement for direct orchidometry. A large body weight can arise from frame size, adiposity, nutritional status, or other somatic factors that are not equivalent to spermatogenic development. By contrast, testicular enlargement is anatomically linked to expansion of seminiferous tubules and development of the germ-cell compartment [3,4,5,8]. In our previously published histology-linked study, mean bilateral testicular volume discriminated bilateral Grade ≥ 4 status with an AUC of 0.976, whereas chronological age and body weight showed AUCs of 0.797 and 0.890, respectively [12]. The present large-scale analysis provides a complementary perspective: body weight adds substantial information beyond chronological age at the population level, but direct testicular measurement remains the more biologically proximal indicator of the developmental state of the organ itself. Together, the two studies support a practical hierarchy of information in which direct orchidometry is the primary screening measure, while age and body weight provide complementary contextual information.

The temporal alignment of body-weight and testicular measurements strengthens this conclusion. Although the source data were collected for routine veterinary purposes rather than a prospective protocol, 97.8% of technically valid weight measurements were obtained on the same day as testicular measurement, and 99.5% were within 30 days. Restricting the analysis to same-day, ±7-day, ±30-day, ±90-day, or ±180-day windows produced highly similar estimates, as did an analysis using interpolated weight where possible. Consequently, the association between body weight and testicular volume is unlikely to be an artifact of the small proportion of records with asynchronous measurement dates. Non-fasted weighing may add short-term biological variability, but such variability would be expected primarily to attenuate rather than create the strong observed association. Moreover, housing and social conditions can transiently affect both body weight and testicular volume in mature cynomolgus macaques, underscoring the need to interpret these measures in their husbandry context [24].

The study has several strengths. The dataset contained more than 31,000 technically valid testicular measurements and more than 22,000 animals, providing substantially greater population coverage than previous orchidometric studies [10,11]. More than 4000 animals had repeated measurements, enabling direct analysis of within-animal developmental change. The use of GEEs accounted for correlation among repeated observations, and sensitivity analyses demonstrated that the body-weight findings were stable across alternative intervals between body-weight and testicular measurements. Importantly, the 8.68 cm^3^ criterion was prespecified from a separately published histology-linked cohort and was not selected, tuned, or optimized from the present population distribution [12]. This separation between threshold derivation and large-scale longitudinal application reduces the risk of circular threshold selection, although the present dataset does not contain histology and therefore does not constitute an independent validation of diagnostic accuracy.

The primary analyses used mean bilateral testicular volume to maintain consistency with the independently derived histology-linked screening threshold. However, bilateral averaging can obscure unilateral size differences that may be relevant during animal selection or reproductive-system evaluation. The supplementary asymmetry analysis showed that marked left–right differences were uncommon but present in a small subset of records. Accordingly, the bilateral mean should be interpreted as a screening summary and should not replace separate clinical assessment of each testis when a unilateral abnormality is suspected. Because the historical dataset did not contain verified diagnostic labels for cryptorchidism or other unilateral lesions, the observed asymmetry cannot be assigned a specific pathological cause. Background testicular lesions with unilateral as well as bilateral distributions have been reported in cynomolgus macaques, further supporting separate clinical assessment of each testis when asymmetry is suspected [25].

Several limitations should nevertheless be considered. First, this was a retrospective analysis of routine health-examination and colony-management records collected over approximately two decades. Measurements were obtained under an institutional SOP using standardized anatomical landmarks and digital calipers; however, individual operator identifiers were not consistently retained throughout the approximately 20-year retrospective period. Consequently, inter-operator effects could not be directly quantified or adjusted for, and residual operator-related measurement variability cannot be excluded. Furthermore, animals with repeated measurements were not prospectively selected for longitudinal follow-up and may not represent a random sample of the overall colony population. The availability and timing of repeated measurements depended on historical colony-management circumstances and the duration for which individual animals remained in the colony. Therefore, the longitudinal transition estimates should be generalized to the broader male cynomolgus macaque population with appropriate caution. In addition, the historical availability of Chinese- and Cambodian-origin animals differed over the approximately 20-year observation period, meaning that geographic origin was partly associated with calendar period. Consequently, residual confounding by temporal changes in husbandry, nutrition, colony management, measurement practices, or operator composition cannot be completely excluded. The origin-related results should therefore be interpreted as comparisons between historical colony populations rather than as estimates of purely genetic effects of geographic origin. Second, external caliper measurements include the practical limitations of measuring through scrotal tissues and do not represent ultrasonographic or postmortem anatomical volume. Third, the present dataset did not contain concurrent histology, semen characteristics, endocrine measurements, or fertility outcomes. In addition, among the technically excluded source records, 80 contained a valid measurement for only one testis while the contralateral measurement was missing, unmeasurable, or non-positive. Because verified diagnostic information explaining these unilateral non-measurements was unavailable, these records could not be classified retrospectively as cryptorchidism, previous orchiectomy, technical measurement failure, or another unilateral condition. Threshold attainment should therefore be interpreted as entry into a range previously associated with bilateral Grade ≥ 4 histology in the published histology-linked cohort [12], not as confirmation of complete adult maturity or fertility in the present animals. Future prospective studies with standardized measurement schedules could use interval-censored time-to-event approaches to estimate threshold-attainment timing more precisely, while mixed-effects models could be used to characterize subject-specific developmental trajectories. Fourth, body weight was measured under routine non-fasted conditions. Fifth, statistical outlier exclusion was based on age- and origin-adjusted residuals; although only 17 records were removed, extreme biological phenotypes cannot be completely distinguished from measurement error in a retrospective dataset. Finally, although the threshold was derived independently from the present dataset, both studies were conducted within the same institution; external validation in other facilities and breeding populations remains necessary before this threshold can be considered broadly generalizable.

From an operational perspective, the results argue against choosing male cynomolgus macaques for reproductive-system evaluation solely by age. An age requirement can enrich a study population for more developed animals, and body weight can further refine that expectation, but both remain indirect. A brief standardized testicular measurement performed during routine physical examination provides individual-specific information at the time of study selection. Such screening may be particularly useful in the 4–6-year age range, where the population transitions rapidly and animals of the same age show the greatest practical uncertainty regarding testicular development.

Future work should prospectively link serial in-life orchidometry with histology, endocrine profiles, semen characteristics, and, where relevant, exposure to known testicular toxicants. Independent validation in other facilities and breeding populations is also needed to assess the transportability of the histology-linked screening threshold. Longitudinal mixed-effects or interval-censored time-to-event approaches could further characterize individual growth trajectories when measurement schedules are prospectively standardized. Such studies would help determine whether the combination of direct orchidometry, age, and body weight can improve animal selection while reducing the likelihood that physiological immaturity complicates interpretation of nonclinical reproductive toxicity.

The present findings also have implications for animal welfare and implementation of the 3Rs. Regulatory and industry discussions have emphasized opportunities to reduce and refine NHP use through improved study design, animal selection, husbandry, and weight-of-evidence approaches in nonclinical and reproductive safety assessment [26,27,28,29,30]. Testicular dimensions can be assessed in life during routine physical examination and may therefore provide useful information before animals are assigned to nonclinical studies, without requiring sacrifice to determine testicular developmental status. Incorporating individual testicular measurements into animal selection may reduce the likelihood of assigning animals with insufficiently developed testes to studies requiring reliable evaluation of the male reproductive system. This approach primarily represents refinement by improving the appropriateness of animal selection and scientific interpretability, and it may also contribute indirectly to reduction by decreasing the risk of inconclusive studies, replacement of unsuitable animals, or additional animal use.

## 5. Conclusions

Longitudinal analysis demonstrated substantial inter-individual variation in testicular development, particularly during approximately 4–6 years of age, such that chronological age alone did not reliably characterize individual developmental status. Body weight provided additional information beyond age for identifying animals within the previously established histology-linked screening range, whereas direct bilateral testicular measurement remained the most biologically proximal practical screening measure. Geographic-origin effects were small relative to the magnitude of age-related developmental change and should be interpreted cautiously given the historical structure of the dataset. Because the 8.68 cm^3^ screening threshold is based on mean bilateral volume, each testis should also be assessed individually when a unilateral abnormality is suspected. Together, these findings support direct testicular measurement as the primary screening approach, with chronological age and body weight serving as complementary information when selecting male cynomolgus macaques for nonclinical studies requiring evaluation of the male reproductive system.

## Figures and Tables

**Table 1:** Descriptive longitudinal attainment of the 8.68 cm^3^ histology-linked screening threshold by geographic origin.

Variable	Cambodian Origin	Chinese Origin
Repeatedly measured animals, n	3072	1259
Initially < 8.68 cm^3^, n	2018	721
Subsequently reached ≥8.68 cm^3^, n	1167	429
Observed transition proportion	57.8%	59.5%
First observed threshold age, median (IQR), years	5.29 (4.70–6.01)	5.72 (5.06–6.40)
Midpoint-estimated transition age, median (IQR), years	4.90 (4.35–5.54)	5.07 (4.56–5.62)
Threshold-spanning interval, median (IQR), years	0.66 (0.47–1.17)	1.18 (0.50–1.94)

Note: Analyses of threshold attainment were restricted to animals whose first valid mean bilateral testicular volume was <8.68 cm^3^. First observed threshold age was defined as the age at the first subsequent measurement with mean bilateral testicular volume ≥ 8.68 cm^3^. Midpoint-estimated transition age was calculated as the midpoint between the last below-threshold measurement and the first at-or-above-threshold measurement. Threshold-spanning interval represents the time between these two measurements. IQR, interquartile range.

**Table 2:** Model-estimated age-dependent testicular development.

Age (Years)	Cambodian: Probability of Volume ≥8.68 cm^3^	Chinese: Probability of Volume ≥8.68 cm^3^	Cambodian Estimated Volume (cm^3^)	Chinese Estimated Volume (cm^3^)
3	2.3%	2.5%	0.99	0.85
4	16.3%	16.2%	3.29	2.97
5	54.3%	51.6%	8.10	7.68
6	84.0%	81.0%	14.83	14.76
7	94.4%	92.6%	20.16	21.07

Note: Estimates are derived from the primary quadratic GEE models. The slight downturn in continuous testicular volume near the upper age boundary is a consequence of the quadratic functional form, was not reproduced in the restricted cubic spline analysis, and is not interpreted as biological regression.

**Table 3:** Primary GEE model for mean bilateral testicular volume ≥ 8.68 cm^3^ and apparent discrimination.

Variable	OR	95% CI	*p* Value
Age_c, per year	1.97	1.84–2.11	<0.001
Age_c^2^	0.85	0.82–0.88	<0.001
Body weight, per 1 kg	4.18	3.93–4.44	<0.001
Chinese vs. Cambodian origin	1.44	1.33–1.56	<0.001
Age_c × Chinese origin	1.40	1.27–1.56	<0.001
Apparent AUC: age + origin	0.896	—	—
Apparent AUC: age + body weight + origin	0.927	—	—

## Data Availability

The de-identified individual-level longitudinal transition data supporting the descriptive analyses are provided in the accompanying Supplementary Dataset (Animals_Article_Supplementary_Data.xlsx), as described in Supplementary Table S5. The complete source colony-management dataset is not publicly available because it contains proprietary operational information. Additional aggregated data supporting the findings of this study may be available from the corresponding author upon reasonable request and subject to institutional approval.

## Supplementary Materials

The following supporting information can be downloaded at: https://www.mdpi.com/article/10.3390/ani16193113/s1, Figure S1: Restricted cubic spline sensitivity analysis of age-dependent mean bilateral testicular volume; Figure S2: Restricted cubic spline sensitivity analysis of the age-dependent probability of attaining the 8.68 cm^3^ histology-linked screening threshold; Table S1: Full coefficients for the longitudinal binary GEE model of attainment of the 8.68 cm^3^ histology-linked screening threshold; Table S2: Full coefficients for the longitudinal Gaussian GEE model of log-transformed mean bilateral testicular volume; Table S3: Sensitivity analyses for the allowable interval between body-weight and testicular measurements; Table S4: Data-cleaning and outlier-exclusion summary for the primary body-weight analysis; Table S5: Individual longitudinal threshold-transition data; Table S6: Descriptive distribution of left–right testicular-volume asymmetry; and Additional Diagnostic Information. The accompanying Supplementary Dataset contains the individual-level longitudinal threshold-transition data described in Table S5. Reference [12] is also cited in the Supplementary Materials.
