# Supplementary material for: Longitudinal Changes in Testicular Volume and the Additional Value of Body Weight for Estimating Histology-Linked Testicular Development in Male Cynomolgus Macaques (Macaca fascicularis)
Source: Animals (Basel). 2026 Oct 3;16(19):3113. doi: 10.3390/ani16193113 (PMC13663561; doi:10.3390/ani16193113)
Supplement: Supplementary file 1 [file animals-16-03113-s001.zip › Supplementary File/Supplementary_Materials_ver2.pdf]

Longitudinal Changes in Testicular Volume and the Additional Value of Body Weight for Estimating Histology-Linked Testicular Development in Male Cynomolgus Macaques (*Macaca fascicularis*)

Supplementary Methods

Prespecified histology-linked threshold. The primary screening threshold was a mean bilateral testicular volume of 8.68 cm^3. This value was prespecified from the independent histology-linked cohort reported by Kawashima et al. (Animals 2026, 16, 2592; doi:10.3390/ani16162592), in which it identified bilateral histological Grade ≥4 with an AUC of 0.976, sensitivity of 87.2%, and specificity of 95.5%. The threshold was not derived or optimized using the present dataset.

Repeated-measures models. For longitudinal analyses restricted to 2–8 years of age, generalized estimating equations (GEE) with an exchangeable working correlation structure and robust standard errors were used to account for repeated observations within animals. The binary model used whether mean bilateral testicular volume was ≥8.68 cm^3 as the outcome with a binomial distribution and logit link. The continuous model used the natural logarithm of mean bilateral testicular volume as the outcome with a Gaussian distribution. Both primary models included age centered at 5 years (age_c = age − 5), the squared centered-age term (age_c²), geographic origin, and an age_c-by-origin interaction.

Restricted cubic spline sensitivity analysis. To evaluate whether the observed age-dependent developmental pattern depended on the quadratic specification of age used in the primary models, sensitivity analyses were performed using restricted cubic splines within the same GEE framework. Four knot locations corresponding to the 5th, 35th, 65th, and 95th percentiles of age in the longitudinal analysis population were used (approximately 2.92, 4.46, 5.50, and 7.39 years, respectively). The binary and continuous outcomes, exchangeable working correlation structure, robust standard errors, and geographic-origin term were retained, and a linear age-by-origin interaction was included.

Body-weight sensitivity analysis. Body weight was measured under routine husbandry conditions without prior fasting. The primary body-weight analysis accepted measurements obtained within ±30 days of the testicular measurement. Sensitivity analyses repeated the models using same-day measurements and allowable intervals of ±7, ±90, and ±180 days. A supplementary interpolation analysis was also performed when suitable body-weight observations were available before and after the testicular measurement.

Data cleaning and outliers. Records lacking one or more dimensions required to calculate both left and right testicular volumes (n = 2,776) and records containing zero or other non-positive testicular dimensions (n = 212) were excluded. Four additional records were excluded as obvious data-entry errors because a single recorded dimension was 85, 86, 313, or 385 mm, whereas the remaining three dimensions in the same record ranged from 8 to 43 mm. These four exclusions were made before statistical modeling. For the primary body-weight analysis, statistical outliers were identified using age- and origin-adjusted residuals of log-transformed body weight and log-transformed mean bilateral testicular volume. An observation was excluded if the absolute residual for either variable exceeded five median absolute deviations from the corresponding residual distribution. Seventeen observations met this statistical outlier criterion.

Bilateral asymmetry analysis. To address potential masking of unilateral differences by the bilateral mean, left–right asymmetry was characterized descriptively across all 31,310 technically valid observations. For each observation, the absolute volume difference was calculated as |V_left − V_right|; the relative bilateral difference was calculated as |V_left − V_right| divided by the bilateral mean and expressed as a percentage; and the larger-to-smaller testicular-volume ratio was calculated. Because the 8.68 cm^3 screening threshold was derived for mean bilateral volume rather than for individual testes, it was not reinterpreted as a unilateral diagnostic cutoff. To assess whether repeated measurements materially influenced the descriptive distribution, the analysis was repeated using only the first valid observation from each animal.

Restricted Cubic Spline Sensitivity Analysis

Figure S1. Restricted cubic spline sensitivity analysis of age-dependent mean bilateral testicular volume.

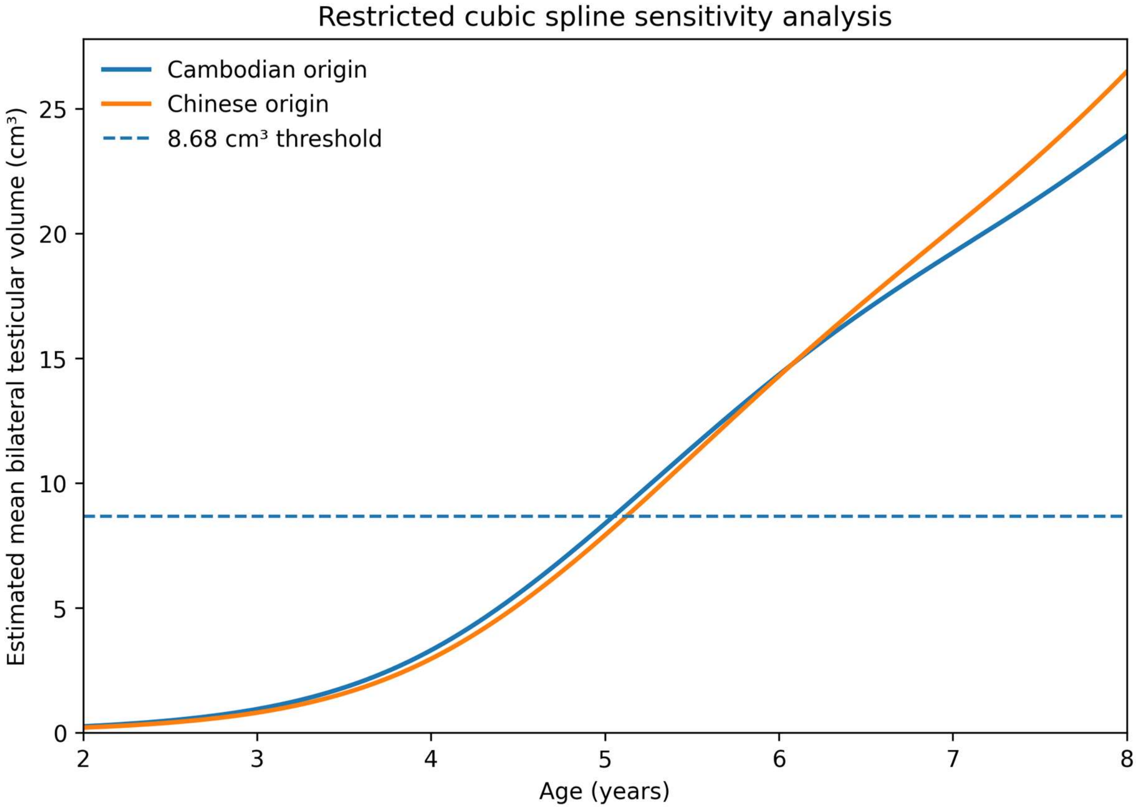

Estimated mean bilateral testicular volume in Cambodian- and Chinese-origin male cynomolgus macaques aged 2–8 years was obtained from Gaussian GEE models of natural-log-transformed testicular volume using restricted cubic splines for age. Four knot locations corresponding to the 5th, 35th, 65th, and 95th percentiles of the observed age distribution were used. The dashed horizontal line indicates the prespecified 8.68 cm^3 histology-linked screening threshold. The spline model reproduced the marked increase in testicular volume during approximately 4–6 years of age but did not reproduce the slight decline toward 8 years predicted by the quadratic model.

Figure S2. Restricted cubic spline sensitivity analysis of the age-dependent probability of attaining the 8.68 cm³ histology-linked screening threshold.

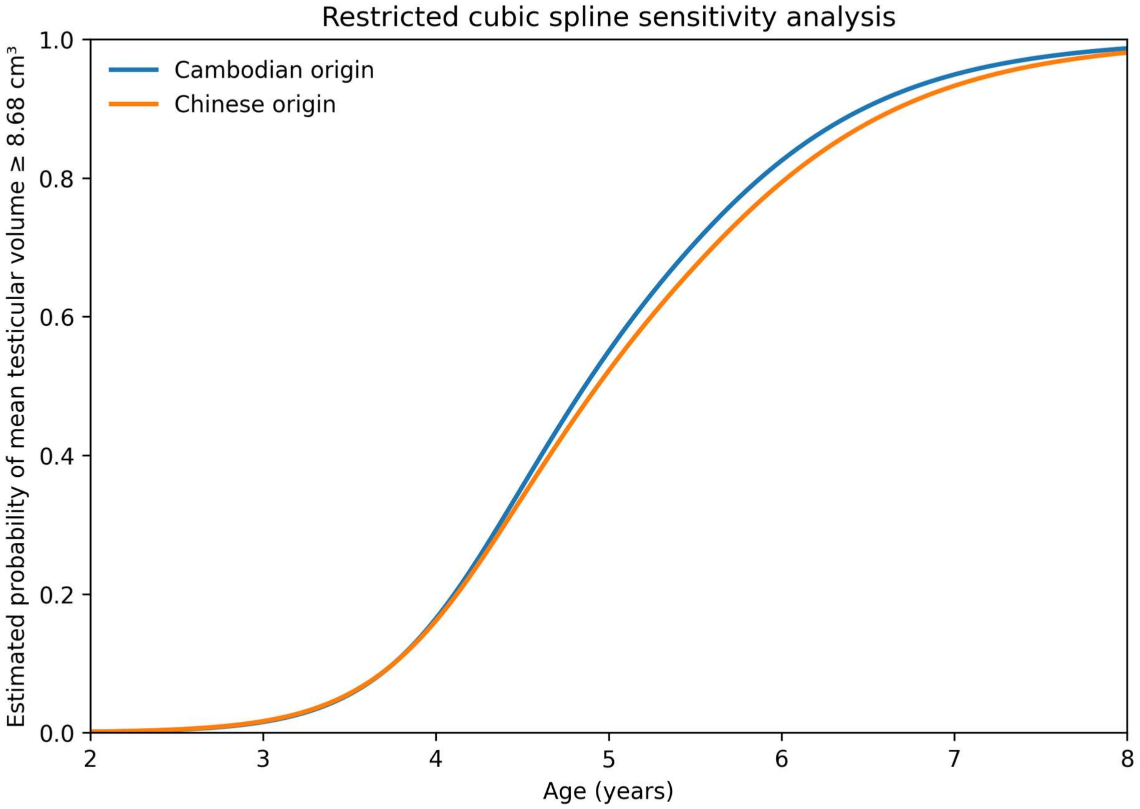

Estimated probabilities were obtained from binomial GEE models in Cambodian- and Chinese-origin male cynomolgus macaques aged 2–8 years using restricted cubic splines for age. Four knot locations corresponding to the 5th, 35th, 65th, and 95th percentiles of the observed age distribution were used. The spline analysis reproduced the marked increase in threshold-attainment probability during approximately 4–6 years of age and the broadly overlapping age-dependent patterns between geographic origins.

RCS sensitivity-analysis summary. The spline models reproduced the principal pubertal developmental pattern observed in the quadratic GEE analyses. At 5 years, estimated threshold-attainment probabilities were 55.0% for Cambodian-origin and 52.2% for Chinese-origin animals; corresponding estimates at 6 years were 82.5% and 79.3%. In the binary spline model, the origin term and age-by-origin interaction were not statistically significant ($p = 0.081$ and $p = 0.230$, respectively). In the continuous-volume spline model, origin-related terms remained statistically significant, but absolute differences during the central pubertal period were small. Unlike the quadratic model, the spline model did not predict a decline in testicular volume toward the upper end of the 2–8-year age range.

Supplementary Tables

Table S1. Full coefficients for the longitudinal binary GEE model of attainment of the 8.68 cm^3 histology-linked screening threshold.

Variable	β	Robust SE	OR	95% CI	*p* value
Intercept	0.1734	0.0386	1.189	1.103–1.283	<0.001
Age_c (centered at 5 y)	1.6466	0.0383	5.189	4.814–5.594	<0.001
Age_c^2	−0.1594	0.0214	0.853	0.818–0.889	<0.001
Chinese vs. Cambodian origin	−0.1088	0.0656	0.897	0.789–1.020	0.097
Age_c × Chinese origin	−0.0989	0.0703	0.906	0.789–1.040	0.160

Outcome: mean bilateral testicular volume ≥8.68 cm^3. Model included 11,451 observations from 4,195 animals aged 2–8 years. GEE used a binomial distribution, logit link, exchangeable working correlation structure, and robust standard errors. Age_c = age − 5 years.

Table S2. Full coefficients for the longitudinal Gaussian GEE model of log-transformed mean bilateral testicular volume.

Variable	β	Robust SE	exp(β)	95% CI for exp(β)	*p* value
Intercept	2.0922	0.0133	8.102	7.893–8.317	<0.001
Age_c (centered at 5 y)	0.7533	0.0076	2.124	2.093–2.156	<0.001
Age_c^2	−0.1487	0.0037	0.862	0.856–0.868	<0.001
Chinese vs. Cambodian origin	−0.0539	0.0232	0.948	0.905–0.992	0.020
Age_c × Chinese origin	0.0490	0.0156	1.050	1.019–1.083	0.002

Outcome: natural logarithm of mean bilateral testicular volume. The same 2–8-year longitudinal population and GEE correlation structure were used as in Table S1. Age_c = age − 5 years.

Table S3. Sensitivity analyses for the allowable interval between body-weight and testicular measurements.

Allowed interval	Obs.	Animals	OR/kg (95% CI)	AUC age	AUC age+BW	Volume multiplier/kg (95% CI)	RMSE age	RMSE age+BW
Same day	28,523	21,881	4.181 (3.928–4.451)	0.896	0.928	1.623 (1.600–1.646)	0.794	0.704
±7 d	28,840	21,905	4.190 (3.938–4.458)	0.896	0.927	1.619 (1.596–1.642)	0.793	0.704
±30 d	29,051	21,932	4.175 (3.926–4.440)	0.896	0.927	1.616 (1.593–1.639)	0.793	0.704
±90 d	29,150	21,937	4.170 (3.922–4.434)	0.895	0.927	1.616 (1.593–1.639)	0.794	0.704
±180 d	29,179	21,940	4.159 (3.911–4.422)	0.895	0.927	1.615 (1.592–1.638)	0.794	0.705

All body-weight coefficients for the binary threshold outcome were statistically significant at $p < 0.001$. Sensitivity analyses used the same GEE model specification and exchangeable working correlation structure as the primary analysis, while varying only the allowable interval between body-weight and testicular measurements. The stability of effect estimates across all timing windows indicates that the small temporal mismatch between body-weight and testicular measurements did not materially affect the conclusions.

Supplementary note: temporal agreement between body-weight and testicular measurements.

Origin	Valid measurements	Same day	Within ±7 d	Within ±30 d	Within ±90 d	Within ±180 d
Cambodian	17,744	17,246	17,448	17,626	17,706	17,735
Chinese	13,566	13,361	13,486	13,543	13,563	13,565
Total	31,310	30,607	30,934	31,169	31,269	31,300

Table S4. Data-cleaning and outlier-exclusion summary for the primary body-weight analysis.

Step	Criterion	Records remaining	Excluded at step
Source population	Chinese- and Cambodian-origin records	34,302	—
Technical validity	Both testes measurable with positive length/width; 2,776 incomplete/unmeasurable records and 212 non-positive records excluded; four isolated implausible values (85, 86, 313, or 385 mm) excluded as obvious data-entry errors	31,310	2,992
Primary analysis window	Age 2–8 years; body weight within ±30 days of testicular measurement	29,068	2,242
Statistical outlier screen	Absolute age- and origin-adjusted residual of log body weight or log volume >5 MAD	29,051	17
Final primary body-weight dataset	Final observations; 21,932 animals	29,051	—

Table S5. Individual longitudinal threshold-transition data.

The complete individual-level dataset is provided in the accompanying Excel file "Animals_Article_Supplementary_Data.xlsx" (sheet: Table S5_Transitions). It contains 2,739 animals that were below 8.68 cm^3 at their first valid observation and had at least one subsequent testicular measurement. Variables include origin, age and mean bilateral testicular volume at first and last measurement, follow-up duration, whether the animal reached the threshold, the last below-threshold measurement, the first at-or-above-threshold measurement, the midpoint-estimated transition age, the threshold-spanning interval, and the corresponding volume increase.

Table S6. Descriptive distribution of left-right testicular-volume asymmetry.

Measure	All valid observations (n = 31,310)	First valid observation per animal (n = 22,305)
Absolute volume difference, median (IQR), cm^3	0.386 (0.075–1.283)	0.272 (0.043–0.908)
Absolute volume difference, 95th percentile, cm^3	4.516	3.445
Relative bilateral difference, median (IQR), %	9.30 (2.99–19.43)	9.37 (2.90–20.66)
Relative bilateral difference, 95th percentile, %	40.78	41.45
Larger/smaller volume ratio, median (IQR)	1.098 (1.030–1.215)	1.098 (1.030–1.230)
Larger/smaller volume ratio, 95th percentile	1.512	1.523
Larger/smaller ratio ≥1.5, n (%)	1,626 (5.19%)	1,208 (5.42%)
Larger/smaller ratio ≥2.0, n (%)	345 (1.10%)	221 (0.99%)
Larger/smaller ratio ≥3.0, n (%)	110 (0.35%)	55 (0.25%)

Note: Testicular volume was calculated as $V = (\pi/6) \times L \times W^2$. Relative bilateral difference = $|Vleft - Vright| / [(Vleft + Vright)/2] \times 100$. The first-observation analysis was performed as a descriptive sensitivity analysis to avoid disproportionate contribution from animals with repeated measurements. These metrics describe bilateral size differences only and do not diagnose cryptorchidism or other unilateral pathology. Among the technically excluded source records, 80 had one measurable testis and a contralateral measurement that was missing, unmeasurable, or non-positive. The historical dataset did not contain verified diagnostic labels that would allow these records to be classified as cryptorchidism, technical measurement failure, or another unilateral condition; therefore, they were not included in the quantitative asymmetry distribution and were not assigned a pathological diagnosis.

Additional Diagnostic Information

Age–body-weight association and multicollinearity. Pearson r = 0.832; Spearman ρ = 0.851. VIFs were 4.33 for age, 1.39 for age^2, 3.31 for body weight, 1.45 for origin, and 2.00 for the age-by-origin interaction.

Body-weight-by-origin interaction. The body-weight-by-origin interaction was not statistically significant for the binary threshold outcome ($p = 0.060$). For continuous log-transformed testicular volume, the interaction was statistically significant ($\exp[\beta] = 1.069$, $p < 0.001$), but the effect magnitude was modest.

Interpolation sensitivity analysis. Among 28,595 eligible measurements in the interpolation check, 28,540 were same-day observations and 55 required linear interpolation. The estimated body-weight OR for the binary threshold outcome was 4.128 per kilogram, and the estimated continuous-volume multiplier was 1.623 per kilogram, consistent with the primary analysis.

Reference for the Prespecified Threshold

Kawashima, R.; Haruyama, E.; Moriyama, A.; Mizuyoshi, K.; Asano, A. In-Life Testicular Volume as an Indicator of Histological Testicular Development Suitable for Testicular Toxicity Evaluation in Male Cynomolgus Macaques (*Macaca fascicularis*). Animals 2026, 16, 2592. https://doi.org/10.3390/ani16162592.
